# Genome Sequences of Three Actinobacteriophage from Cluster FH Isolated Using Arthrobacter globiformis

**DOI:** 10.1128/mra.00805-22

**Published:** 2022-10-27

**Authors:** Shannon Isenhart, Adam Gillison, Darien Brown, Auremie Kleven, Macayla Allen, Brittany Bohn, Lee Anne Martínez, Amaya García Costas

**Affiliations:** a Department of Biology, Colorado State University—Pueblo, Pueblo, Colorado, USA; Queens College CUNY

## Abstract

We report the discovery and genome sequences of three FH cluster actinophage infecting Arthrobacter globiformis B2979. Lilmac1015 and Klevey were isolated from riverbank soil and Prairie from soil collected below a tree. Their respective genome lengths are 49,978, 50,075, and 49,392 bp, with 80, 81, and 78 predicted protein-coding genes.

## ANNOUNCEMENT

Bacteriophage are the most numerous biological entities on Earth known thus far. Their astounding ecological dominance and genetic diversity have been realized recently, with many studies revealing new clades of phage from a diversity of environments (1–5). Here, we present the isolation and genome sequences of three actinophage that are members of a newly defined cluster (https://phagesdb.org/).

**FIG 1:**
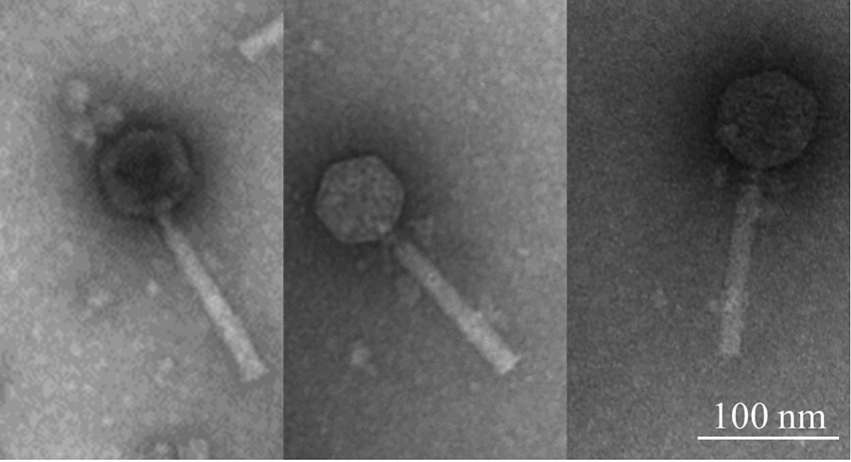
Transmission electron micrographs of siphovirus phage Prairie, Klevey, and Lilmac1015 (left to right). A high-titer (>1.0 × 10^5^ PFU/mL) lysate of each phage was negatively stained with 1% uranyl acetate.

Klevey and Lilmac1015 were isolated from soil samples collected next to the Arkansas River in Pueblo, CO (38.2682 N, 104.6767 W), and Fowler, CO (38.157415 N, 104.079396 W), respectively; Prairie was isolated from prairie soil under a tree in Pueblo, CO (38.574566 N, 104.684261 W). Lilmac1015 and Prairie were isolated directly from soil slurry as outlined in the SEA-PHAGES manual (6); Klevey generated plaques following a 2-day incubation with the host of the soil slurry. All phage infected the host Arthrobacter globiformis B2979 in peptone-yeast-calcium (PYCa) medium at 30°C. All formed uniform circular plaques that were clear during the first 48 h but with visible cloudiness after longer incubations. The purified plaques were used for microscopy and genomic analyses. Transmission electron microscopy analyses revealed all three to have an icosahedral head and noncontractile flexible tail (Fig. 1), characteristic of siphoviruses (7).

Genomic DNA extraction was performed at Colorado State University—Pueblo using Promega’s Wizard DNA extraction kit following the manufacturer’s instructions and the SEA-PHAGES manual (6). The sequencing libraries were prepared at the Pittsburgh Bacteriophage Institute using a NEBNext Ultra II FS kit with dual indexed barcoding and sequenced using an Illumina MiSeq platform, yielding 67,799 (Lilmac1015), 151,385 (Klevey), and 649,437 (Prairie) 150-bp single-end reads. The raw reads were assembled using Newbler 2.9, with default settings, into single phage contigs with approximate shotgun coverages of 194-, 436-, and 1,874-fold, respectively; the contigs were checked for completeness, accuracy, and phage genomic termini using Consed 29, as described previously (8, 9). All three genomes are circularly permuted, with lengths of 49,978, 50,075, and 49,392 bp, and G+C contents of 69.4%, 69.4%, and 69.5% (Lilmac1015, Klevey, and Prairie, respectively).

The genome sequences were automatically annotated using Glimmer (10) and GeneMark (11) and subsequently manually curated using DNA Master (12), Phamerator (13), and Starterator (14). Functions for each coding sequence were assigned based on the top hits from searches using NCBI blastp (15), PhagesDB blastp (16), and HHpred (17). Membrane proteins were identified using TMHMM 2.0 (18) and SOSUI (19). All tools were run with default parameters.

Lilmac1015, Klevey, and Prairie are predicted to have 80, 81, and 78 protein-coding genes, respectively. Only 32 (40%), 29 (35.8%), and 27 (34.6%) of these genes have putative functions, respectively, whereas 48 (60%), 52 (64.2%), and 51 (65.4%) have no known function. All three bacteriophage have a gene that codes for ParB-like nuclease domain protein, indicating that they may be temperate phage, though no repressor has been identified.

Using the nucleotide sequence of each phage as the query for a BLASTn search against the NCBI nonredundant (nr) database revealed that all three phage belong to the newly designated actinophage cluster FH. Klevey and Prairie are the most closely related, with 96% shared identity, whereas Lilmac1015 shares 95% and 94% identity with each of these phage, respectively.

### Data availability.

The complete genome sequences of Lilmac1015, Klevey, and Prairie have been deposited at GenBank under accession numbers OL742560, MZ747522, and MW601223 and SRA accession numbers SRX15605403, SRX15605401, and SRX15605405, respectively. The BioProject accession number is PRJNA488469.
